# Surgical Delay Beyond Three Months for Primary Anterior Cruciate Ligament Reconstruction Does not Increase Risk of Medial Meniscal Ramp Lesions or Lateral Root Tears

**DOI:** 10.1016/j.asmr.2025.101292

**Published:** 2025-10-22

**Authors:** Sandra Wan, W P Yau

**Affiliations:** Department of Orthopaedics and Traumatology, School of Clinical Medicine, Li Ka Shing Faculty of Medicine, The University of Hong Kong, Hong Kong, China

## Abstract

**Purpose:**

To report the percentages of full-thickness meniscal tears per anterior cruciate ligament reconstruction (ACLR) at the time of primary ACLR and the contributions of individual tear patterns, specifically ramp lesions and root tears, to the overall prevalence of tears.

**Methods:**

This retrospective cross-sectional study included patients from 2007 to 2021. The inclusion criteria were patients who underwent primary ACLR. Patients were excluded if they had suffered from multiligamentous injuries, were skeletally immature, or had missing information regarding the outcomes. The outcomes included the presence of full-thickness meniscal tears detected during arthroscopy, the tear pattern according to the International Society of Arthroscopy, Knee Surgery, and Orthopaedic Sports Medicine classification, and the time between injury and ACLR. Follow-up data were not collected.

**Results:**

Among 731 patients, 606 were male, and 125 were female. The mean age was 28 ± 8 years, with an average time between injury and surgery of 539 ± 988 days. The primary causes of injury were soccer (38%) and basketball (32%). Medial meniscal (MM) tears occurred in 43.7% knees, while lateral meniscal (LM) tears occurred in 47.5% of patients. Ramp lesions accounted for 46.9% of all MM tears, and root tears accounted for 15.8% of all LM tears. As the time from injury to ACLR increased, MM tears significantly increased (*P* < .001). However, the time between injury and ACLR was not able to discriminate between the presence or absence of lateral meniscus tears (*P* = 0.84), ramp lesions (*P* = .06), or root tears (*P* = .16).

**Conclusions:**

Aside from the initial 3 months postinjury, the percentage of medial meniscal tears per ACLR steadily increases with increasing time elapsed from injury to surgery, whereas the percentage of lateral meniscal tears per ACLR remains unchanged. Increased surgical delay is not associated with a higher chance of ramp lesions or lateral meniscus root tears observed at the time of primary ACLR.

**Level of Evidence:**

Level III: Retrospective cross-sectional study.

There has been a rapid increase in the number of publications about meniscal tears at the time of anterior cruciate ligament reconstruction (ACLR) over the past 10 to 15 years, particularly those concerning ramp lesions of the medial meniscus^1^, ^2^, ^3^, ^4^, ^5^, ^6^, ^7^, ^8^, ^9^ and root tears of the lateral meniscus.^10^, ^11^, ^12^, ^13^, ^14^, ^15^, ^16^, ^17^, ^18^, ^19^ Ramp lesions are commonly defined as longitudinal tears of the red–red zone of the posterior horn of the medial meniscus and may involve disruption of the meniscocapsular junction or meniscotibial ligament.^20^ This disruption may be associated with a loss of the stabilizing function of the medial meniscus against anterior translation, especially in the scenario of ACL deficiency,^21^^,^^22^ which may potentially increase the chance of graft rupture in patients who undergo ACLR. On the other hand, root tears are meniscal tears that occur at the root of the meniscus or within 9 mm of the root.^14^ Over 85% of the root tears are complete radial tears of the lateral meniscus.^23^ A complete root tear is believed to result in a loss of hoop stress within the meniscus and subsequent meniscus extrusion, leading to a higher chance of early osteoarthritis.^24^^,^^25^ The reported rate of meniscal tears found at the time of primary ACLR ranged from 9% to 41.7% for medial meniscal ramp lesions^2^^,^^4^^,^^26^ and 5.1% to 33.8% for lateral meniscal root tears.^24^^,^^27^

Longitudinal tears are the most common tear pattern of meniscal tears found at the time of ACLR, regardless of whether it is the medial meniscus or the lateral meniscus.^28^, ^29^, ^30^ The reported rate of longitudinal tears ranges from 11.7% to 41.2%.^30^, ^31^, ^32^, ^33^, ^34^, ^35^ On the other hand, radial tears of the lateral meniscus have been historically considered uncommon, with a reported prevalence of less than 2%.^30^ While ramp lesions are a type of longitudinal tear,^20^ and most root tears are radial tears,^23^ given that the average pooled reported rates of ramp lesions and root tears in systematic reviews can be as high as 21.9%^36^ and 9.6%,^37^ respectively, it is possible that some of the previous literature^30^, ^31^, ^32^, ^33^, ^34^, ^35^ has under-reported the rate of medial and lateral meniscal tears due to the omission of the ramp and root lesions.

In addition, despite the large number of publications relating to ramp lesions^9^^,^^33^^,^^38^^,^^39^ or root tears,^10^^,^^11^^,^^13^^,^^24^^,^^27^^,^^40^ few studies have quantified the contribution of these specific tear patterns to the overall prevalence of medial and lateral meniscal tears found at primary ACLR.^31^^,^^33^, ^34^, ^35^ There is a need to update the current literature by providing a detailed report of the relative contributions of individual tear patterns to the overall prevalence of meniscal tears observed at the time of ACLR.

The purpose of this study was to report the percentages of full-thickness meniscal tears per ACLR at the time of primary anterior cruciate ligament reconstruction and the contributions of individual tear patterns, specifically ramp lesions and root tears, to the overall prevalence of tears. The hypotheses are (1) there is no difference in the proportion of specific meniscal tear patterns between the medial and lateral meniscus; and (2) there is no association between the presence of a meniscal tear, including ramp lesions and root tears, and the time between injury and ACLR.

## Methods

This is an Institutional Review Board (IRB)-approved retrospective cohort study based on prospectively collected data for patients who underwent ACLR at a single institute from July 1, 2007, to December 31, 2021. The current study was approved by the local ethic committees: IRB of the University of Hong Kong / Hospital Authority Hong Kong and West Cluster (HKU/HA HKW IRB; approval document number: UW 25-192). The data were prospectively documented using a standard research documentation form, designed for the use in other studies conducted at the author’s institute. The need to obtain informed consent from the participants was waived by the local ethics committee. Patients were included in the study if they underwent primary ACLR. Patients were excluded if (1) they suffered from multiligamentous knee injuries; (2) they were skeletally immature at the time of ACLR; (3) there was missing information regarding the time between injury and surgery; or (4) there was missing information regarding the pattern of meniscal tears.

### Preoperative Assessment

Patients were assessed in a preoperative assessment clinic 1 week prior to their scheduled surgery. Demographic data, including age, sex, as well as information such as the preinjury Tegner Activity Scale (TAS), the type of sport or activity that led to the injury, and the time between injury and ACLR. Activity leading to injury was classified according to the IKDC classification into very strenuous, strenuous, moderate, and light.^41^

### Surgical Procedure

The surgeries were performed by one of the two sports medicine surgeons, including the author (W.P.Y.). Patients were placed under general anaesthesia in a supine position. A pneumatic tourniquet was applied to the proximal thigh and inflated to a pressure of 250 mm Hg. Standard anterolateral and anteromedial portals were created. Diagnostic arthroscopy was then performed with a 30° arthroscope. The site and pattern of the full-thickness tear of the meniscus were documented by the surgeon, according to the International Society of Arthroscopy, Knee Surgery, and Orthopaedic Sports Medicine (ISAKOS) classification. Treatment of the meniscal tears was then performed before ACLR.

### Study Outcomes

The primary outcome of this study was the presence of full-thickness medial and lateral meniscal tears at the time of diagnostic arthroscopy. Knees were classified as having a medial or lateral meniscal tear based on the presence of a tear in the respective area, regardless of any tear in the opposite meniscus. Knees with meniscal tears were defined as knees having a full-thickness meniscal tear in either the medial, lateral, or both menisci. The secondary outcomes were (1) the site of tear, (2) the pattern of tear, and (3) the time between injury and ACLR. The site of the tear was recorded according to whether it was in the anterior horn, body, posterior horn, or a combination of the aforementioned. Tear patterns were categorized according to a modification of the ISAKOS classification, into longitudinal tears (including bucket-handle tears but excluding ramp lesions), horizontal tears, radial tears (excluding root tears), ramp lesions (if it was the medial meniscus), root tears, and other tears, which are generally irreparable (complex tears, beak tears, flap tears, degenerative tears, and meniscal tears with tissue loss). Ramp lesions were defined as longitudinal lesions occurring at the red–red zone of the posterior horn of the medial meniscus.^9^ Root tears were defined as tears located at the root of the meniscus or within 9 mm from the root, involving either the anterior or posterior horns of the meniscus.^14^ All this information was prospectively documented at the time of surgery using a standardized research documentation form completed by the surgeons. In addition, the number of ramp lesions and root tears were reported according to the Sonnery-Cottet^20^ and LaPrade^14^ classification systems, respectively. For cases performed before the publication of these studies (i.e., before 2016), the subtypes of ramp lesions and root tears were retrospectively classified by reviewing operative videos and medical records. Starting from 2016, data regarding the subtypes were prospectively documented alongside other information during the procedures. Time between injury and ACLR was recorded in days and was subsequently grouped into the following intervals: 0 to 3 months, 4 to 6 months, 7 to 12 months, 13 to 24 months, 25 to 60 months and >60 months.^42^, ^43^, ^44^

### Data Analysis

Descriptive data regarding the demographic data, the cumulative total of ACLRs, the number of meniscal tears, including the site and pattern of the tears, and the time between injury and ACLR were reported. The percentage of meniscal tears per ACLRs were provided. TAS and the most common sport activities leading to the injury were presented. The contribution of ramp lesions to the medial meniscal tears is calculated as the proportion of the number of ramp lesions in relation to the number of medial meniscal tears (%). The same method is applied for root tears and lateral meniscal tears. The percentage of medial and lateral meniscal tears per ACLR were compared using the χ^2^-test. Subsequently, the percentage of specific tear patterns per ACLR were compared between the medial and lateral meniscus using the χ^2^-test. The relationship between the rate of meniscal tears and the time between injury and ACLR (in days) was examined using Pearson correlation. The strength of correlation is considered weak when the r value is between 0.1 and 0.3, moderate between 0.3 and 0.5, strong between 0.5 and 0.7, and very strong between 0.7 and 1. Logarithmic transformation was applied to the time between injury and ACLR. The relationship between the percentage of meniscal tears per ACLRs and time was visualized using scatterplots. If a significant association was identified using Pearson correlation, receiver operating characteristic analysis was conducted to evaluate the ability of the time (in days) to discriminate between the presence and absence of a meniscal tear. Linear regression analysis was performed to examine the change in the percentage of meniscal tears per ACLR over time, and *R*^2^ was reported to represent the proportion of variance in the dependent variable explained by time in the regression model. Standardized β was reported. Additionally, time was also categorized into discrete intervals: 0–3 months, 4–6 months, 7–9 months, 10–12 months, 13–24 months, 25–60 months, and >60 months. The acute, subacute, and chronic settings were defined as 0-3 months, 4-12 months, and >12 months, respectively. The relationship between the percentage of meniscal tears per ACLR and the aforementioned time periods was assessed using the χ^2^ test. The difference in the time from injury to ACLR between patients with medial and lateral meniscal tears was analyzed using Kaplan–Meier survival analysis, with the log-rank, Breslow, and Tarone-Ware tests to assess statistical significance. The relationship between the proportion of ramp lesions among medial meniscal tear and time was examined using scatterplots. The same approach was applied to analyze the proportion of root tears among lateral meniscal tears. *P* < .05 was defined as statistically significant. Finally, the data were disaggregated by sex and reported.

### Power Analysis

Erard et al.^31^ reported on meniscal tears, according to the ISAKOS classification, which does not specifically include ramp lesions. The reported percentage of longitudinal tears per ACLR, including bucket-handle tears, in the medial meniscus was 21.6%.^31^ Giurazza et al.^33^ reported the percentage of medial meniscal tears per ACLR classified as vertical lesions, bucket-handle lesions, ramp lesions, flap lesions, and complex lesions. The combined percentage for vertical lesions, bucket-handle lesions, and ramp lesions per ACLR, as reported by Giurazza et al.,^33^ was 32.5%. To detect a clinically important increase of 10.9% by including the ramp lesions in the classification of meniscal tears, with a power of 80% and an α of 0.05, a minimum of 520 knees were required.

## Results

**Fig. 1:**
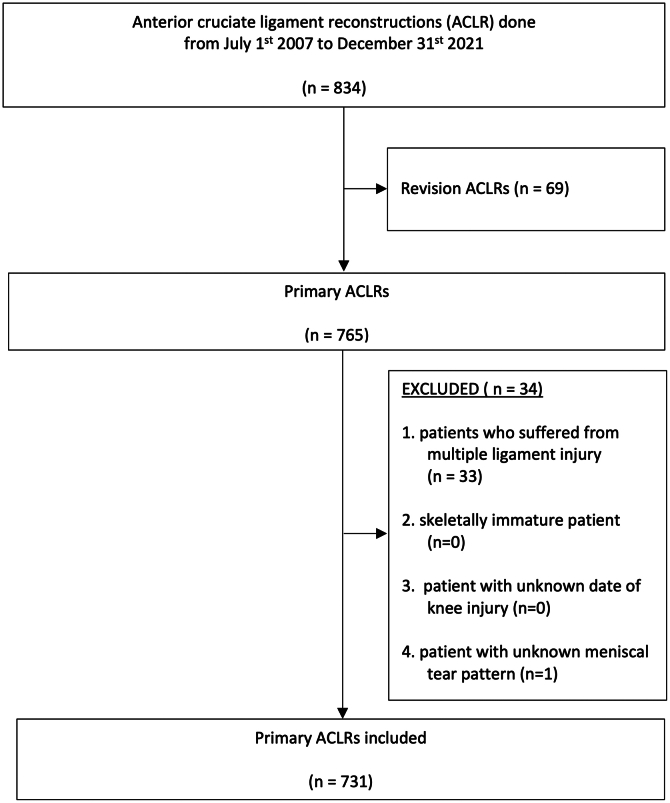
Enrolment of subjects. ACLR, anterior cruciate ligament reconstruction; n, number.

731 patients were included in the analysis (Fig 1). 606 (83%) were male and 125 (17%) were female, with an average age of 28 ± 8 years. The time between injury and ACLR was 539 ± 988 days, with a median of 204 days and an interquartile range from 109 to 492 days. The preinjury Tegner activity scale was 6.5 ± 1.3. Ninety-one percent of patients were injured while participating in a pivoting sport. The most common activity leading to the injury was soccer (38%), followed by basketball (32%).

### Site of Meniscal Tear

Meniscal tears were found in 508 knees, accounting for 69.4% of primary ACLR. Of the 731 knees, 320 (43.7%) had medial and 348 (47.5%) had lateral meniscal tears; these percentages did not differ significantly (*P* = .208). Regarding the tear site, tears most commonly occurred at the posterior horn in isolation (*P* < .001), followed by the body and the posterior horn (*P* < .001) (Table 1).

**Table 1:** Sites of Meniscal Tears

Site of Tear	Medial Meniscus	Lateral Meniscus
Anterior horn	3	5
	0.4%	0.7%
Body	8	26
	1.1%	3.5%
Posterior horn	201	247
	27.5%	33.8%
Body + posterior horn	96	59
	13.1%	8%
Anterior horn + body + posterior horn	8	7
	1.1%	1%
Anterior horn + body	1	1
	0.1%	0.1%
Anterior horn + posterior horn	3	3
	0.4%	0.4%

% refers to the percentage in relation to the total number of anterior cruciate ligament reconstructions.

### Pattern of Meniscal Tears

**Fig. 2:**
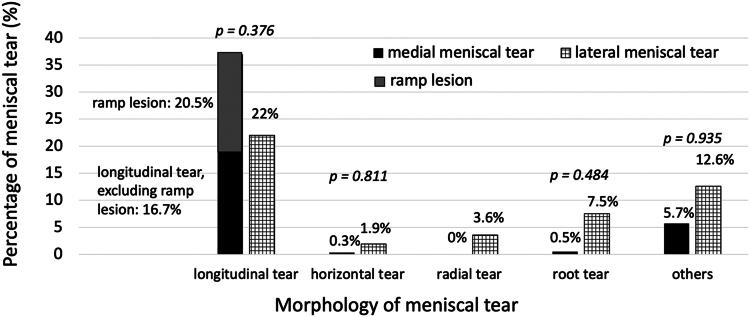
Comparison of tear patterns between medial and lateral menisci. Radial tears do not include root tears, while other tears include beak tears, flap tears, complex tears, degenerative tears, and tears with loss of meniscus tissue. % refers to the percentage rates of meniscal tear in relation to all anterior cruciate ligament reconstructions. *P* is the value of the statistical comparison of the corresponding tear pattern between medial and lateral meniscus.

The distribution of tear patterns relative to the medial or lateral meniscus is shown in Fig 2. The percentage of different tear patterns per ACLR was comparable between the medial and lateral menisci (Fig 2). Out of 731 knees, 122 were found to have longitudinal tears (excluding ramp lesions) (16.7%), and 150 had ramp lesions (20.5%) in the medial meniscus. With regard to the lateral meniscus, 161 longitudinal tears (22%), 55 root tears (7.5%) and 26 radial tears (3.6%) were found.

Among the 150 ramp lesions, according to the Sonnery-Cottet classification,^20^ 15% were type 1, 5% were type 2, 3% were type 3, 69% were type 4, and 8% were type 5. Regarding the 55 root lesions of the lateral meniscus, 2 were anterior horn root tears and 53 were posterior horn root tears. According to the LaPrade classification,^14^ 30.9% were type I, 25.5% were type II, 9.1% were type III, 32.7% were type IV, and 1.8% were type V.

### Relationship Between Meniscal Tears and Time Between Injury and Surgery

**Fig. 3:**
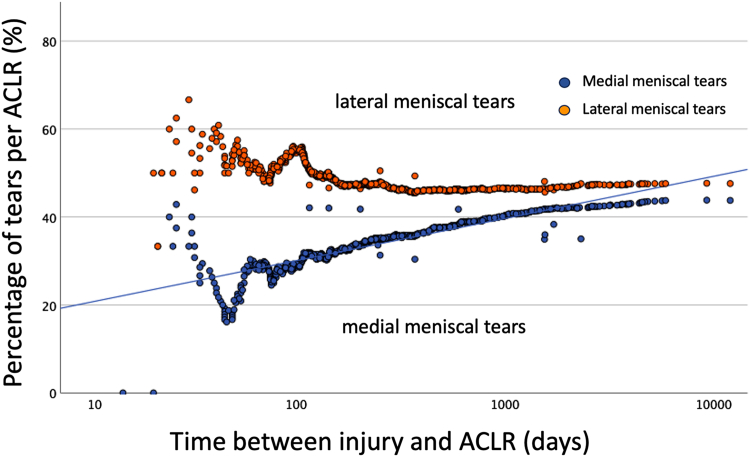
Comparison of the percentages of medial and lateral meniscal tears per anterior cruciate ligament reconstruction (ACLR) in relation to time between injury and ACLR.

Figure 3 depicts the relationship between the percentage of meniscal tears per ACLR and the time elapsed between injury and ACLR, as well as the comparison of the rates of medial and lateral meniscal tears over time as a continuous variable. The relationship between meniscal tears and the time elapsed from injury to ACLR in days is reported in Table 2. There was a moderate to strong positive correlation between the percentage of medial meniscal tears per ACLR and the time elapsed from injury to ACLR. Weak correlations were observed between time and the percentages of lateral meniscal tears, ramp lesions, and root tears per ACLR. When treating time as a continuous variable, it was able to distinguish the presence or absence of medial meniscal tears, but not for the other lesion types. The percentage of medial meniscal tear per ACLR increased with time (*p* < .001), accounting for 33% of the variance (Table 2). In contrast, the percentage of lateral meniscal tear per ACLR was high initially. It began to decrease around 100 days after the initial injury leading to the ACL tear. Subsequently, the tear percentage remained stable despite further increases in the time between injury and ACL reconstruction (Fig 3). A significant difference was observed between the percentages of medial and lateral meniscal tears per ACLR (Breslow test, *P* = .006; Tarone-Ware test, *P* = .029) (Fig 3).

**Table 2:** The relationship between meniscal tears and the time between injury and ACLR (in days).

	Medial Meniscal Tears	Lateral Meniscal Tears	Medial Meniscus Ramp Lesions	Lateral Meniscus Root Tears
Pearson correlation	r = 0.57 (P < .001∗)	r = −0.24 (P < .001∗)	r = 0.33 (P < .001∗)	r = −0.13 (P < .001∗)
ROC analysis	AUC = 0.64 (P < .001∗)	AUC = 0.50 (P = .84)	AUC = 0.55 (P = .06)	AUC = 0.56 (P = .16)
Regression analysis	*R*^2^ = 0.33	*R*^2^ = 0.06	*R*^2^ = 0.11	*R*^2^ = 0.13
	standardized beta = 0.57∗	−	−	−

AUC, area under curve; r, correlation coefficient; ROC, receiver operating characteristics analysis; *R*^2^, proportion of variance in the dependent variable explained by the regression model. An asterisk denotes statistical significance. – indicates not performed.

**Fig. 4:**
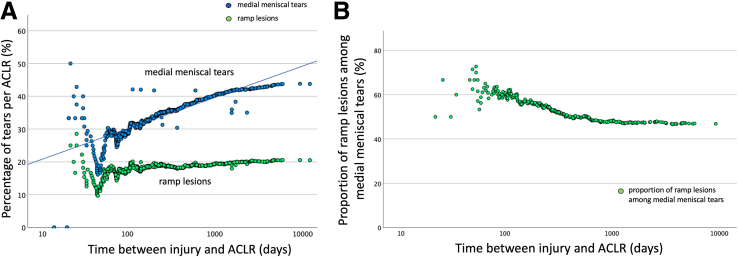
Contribution of ramp lesion to medial meniscal tear. (A) Comparison of the percentages of medial meniscal tears and ramp lesions in relation to the time between injury and anterior cruciate ligament reconstruction (ACLR). (B) Proportion of ramp lesions among medial meniscal tears in relation to the time between injury and ACLR.

Figure 4 depicts the relation between ramp lesions and medial meniscal tears in relation to the time between injury and ACLR.

**Fig. 5:**
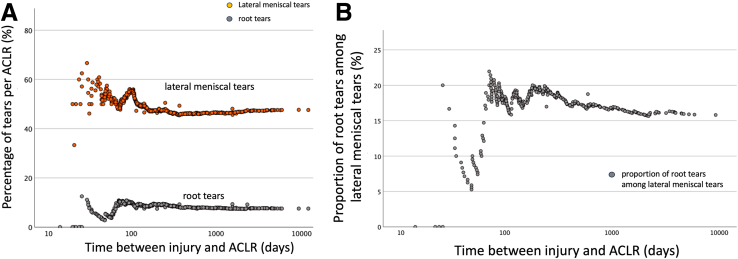
Contribution of root tears to lateral meniscal tears. (A) Comparison of the percentages of lateral meniscal tears and root tears per anterior cruciate ligament reconstruction (ACLR) in relation to the time between injury and ACLR. (B) Proportion of root tears among lateral meniscal tears in relation to the time between injury and ACLR.

There were a total of 150 ramp lesions, accounting for 20.5% of the ACLRs (Table 3). The percentage of ramp lesions per ACLR increased to a peak around 100 days and then remained relatively unchanged over time (Table 3). Ramp lesions accounted for 46.9% of the observed total number of medial meniscal tears (Fig 4B). The proportion of ramp lesions among medial meniscal tears was highest (73%) during the second and third month after injury. Subsequently, there was a progressive decline in the contribution of ramp lesions to the medial meniscal tears, decreasing to 46.9% over time. Figure 5 depicts the relation between root tears and lateral meniscal tears in relation to the time between injury and ACLR.

**Table 3:** Distribution of Tear Patterns in Relation to the Medial or Lateral Meniscus and Time Between Injury and ACLR

	Total	0-3 Months	4-6 Months	7-9 Months	10-12 Months	13-24 Months	25-60 Months	>60 Months	P Value
Number of knees	**731**	140	199	103	59	103	77	50	
**Knees with medial meniscal tears**	**320**	40	71	44	24	56	51	34	P < .001∗
	**43.7%**	28.6%	35.7%	42.7%	40.0%	54.4%	66.2%	68.0%	
Longitudinal tears	**122**	12	25	22	12	23	16	12	P = .017∗
	**16.7%**	8.6%	12.6%	21.4%	20.0%	22.3%	20.8%	24.0%	
Horizontal tears	**2**	0	0	0	0	1	1	0	P = .40
	**0.3%**	0.0%	0.0%	0.0%	0.0%	1.0%	1.3%	0.0%	
Radial tears	**0**	0	0	0	0	0	0	0	--
	**0.0%**	0.0%	0.0%	0.0%	0.0%	0.0%	0.0%	0.0%	
Ramp lesions	**150**	24	38	20	9	23	22	14	P = .33
	**20.5%**	17.1%	19.1%	19.4%	15.0%	22.3%	28.6%	28.0%	
Root lesions	**4**	1	1	0	1	0	1	0	P = .71
	**0.5%**	0.7%	0.5%	0.0%	1.7%	0.0%	1.3%	0.0%	
Other tears	**42**	3	7	2	2	9	11	8	P < .001∗
	**5.7%**	2.1%	3.5%	1.9%	3.3%	8.7%	14.3%	16.0%	
**Knees with lateral meniscal tears**	**348**	75	85	47	21	52	39	29	P = .086
	**47.5%**	53.6%	42.7%	45.6%	35.0%	50.5%	50.6%	58.0%	
Longitudinal tears	**161**	35	39	28	11	22	20	6	P = .24
	**22.0%**	25.0%	19.6%	27.2%	18.3%	21.4%	26.0%	12.0%	
Horizontal tears	**14**	0	2	0	3	0	3	6	P < .001∗
	**1.9%**	0.0%	1.0%	0.0%	5.0%	0.0%	3.9%	12.0%	
Radial tears	**26**	7	6	1	0	8	1	3	P = .07
	**3.6%**	5.0%	3.0%	1.0%	0.0%	7.8%	1.3%	6.0%	
Root lesions	**55**	14	17	8	2	7	3	4	P = .54
	**7.5%**	10.0%	8.5%	7.8%	3.3%	6.8%	3.9%	8.0%	
Other tears	**92**	19	21	10	5	15	12	10	P = .45
	**12.6%**	13.6%	10.6%	9.7%	8.3%	14.6%	15.6%	20.0%	

Longitudinal tears include bucket-handle tears, excluding ramp lesions; radial tears exclude root tears. Other tears include flap tears, complex tears, degenerative tears, and meniscal loss. % refers to the percentage rates refer to the rates of meniscal tear in relation to the number of anterior cruciate ligament reconstructions performed at that time interval. ∗Statistically significant when the P value is <.05.

There were a total of 55 root tears, accounting for 7.5% of the ACLRs (Table 3). Root tears were not observed when ACLR was performed within the first 3 weeks after injury. Subsequently, the percentage of root tears per ACLR fluctuated, reaching a peak of 10% when the time between injury and ACLR was around 3 months and remained relatively unchanged despite further increases in the time between injury and ACLR (Figure 5A). Root tears contributed to 15.8% of all the observed lateral meniscal tears. Except for the first 3 months, the ratio of lateral meniscal root tears to lateral meniscal tears showed minimal change relative to the time between injury and ACLR.

### Data Disaggregation According to Sex

**Fig. 6:**
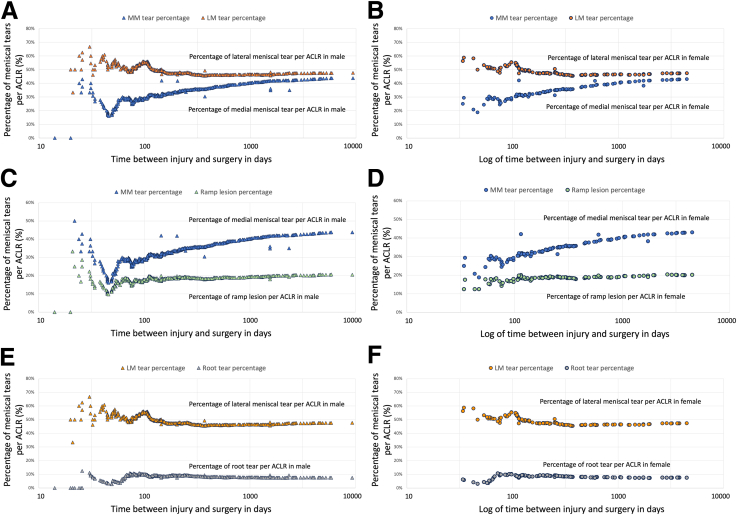
Comparison of the percentages of meniscal tears, ramp lesions, and root tears per anterior cruciate ligament reconstruction (ACLR) between male and female patients. (A) Comparison of the percentages of medial and lateral meniscal tears per ACLR in male patients. (B) Comparison of the percentages of medial and lateral meniscal tears per ACLR in female patients. (C) Comparison of the percentages of medial meniscal tears and ramp lesions per ACLR in male patients. (D) Comparison of the percentages of medial meniscal tears and ramp lesions per ACLR in female patients. (E) Comparison of the percentages of lateral meniscal tears and root tears per ACLR in male patients. (F) Comparison of the percentages of lateral meniscal and root tears per ACLR in female patients. LM tears, lateral meniscal tears MM tears; medial meniscal tears.

The data are presented in Table 5 and Figure 6. The distribution of the site and pattern of meniscal tears in the medial and lateral menisci was comparable between males and females (Table 5). There was no difference between male and female patients regarding the comparison of the percentages of medial and lateral meniscal tears per ACLR (Fig 6, A and B), the percentages of medial meniscal tears and ramp lesions (Fig 6, C and D), and the percentages of lateral meniscal tears and root tears (Fig 6, E and F).

**Table 5:** Distribution of sites and Morphology of Meniscal Tears According to Sex

	Medial Meniscal Tears		Lateral Meniscal Tears	
	Male	Female	Male	Female
**Sites of tear**				
Anterior horn	0.2%	1.6%	0.8%	0%
Body	1.2%	0.8%	3.6%	3.2%
Posterior horn	27.6%	27.2%	35.1%	27.8%
Body + posterior horn	13.2%	12.8%	8.9%	4.0%
Anterior horn + body + posterior horn	1.3%	0%	1.0%	0.8%
Anterior horn + body	0.2%	0%	0.2%	0%
Anterior horn + posterior horn	0.5%	0%	0.5%	0%
**Morphology of tear**				
Longitudinal tear, excluding ramp lesion	16.5%	17.4%	23.4%	14.4%
Ramp lesion	20.6%	19.8%	n/a	n/a
Horizontal tear	0.3%	0%	2.3%	0%
Radial tear	0%	0%	3.8%	2.4%
Root tear	0.7%	0%	7.8%	6.4%
Others	5.8%	4.8%	12.8%	5.6%

% refers to percentage in relation to the total number of anterior cruciate ligament reconstructions; others denotes morphology of tears, other than longitudinal tear, horizontal tear, radial tear, and root tear. n/a, not available.

## Discussion

The most important findings of this study were that: (1) the percentage of medial meniscal tears per ACLR increased with the increasing time between injury and ACLR; (2) while the percentage of lateral meniscal tears per ACLR was high initially, it began to decrease when the time between injury and ACL reconstruction was around 100 days and, subsequently, remained steady despite further surgical delays; (3) beyond the first 100 days after injury, the percentages of both ramp lesions and lateral meniscus root tears per ACLR remained unchanged over time; and (4) the proportion of ramp lesions among the medial meniscal tears was higher within the first 3 months from injury, compared with subsequent time points, whereas the proportion of root injuries among lateral meniscal tears fluctuated within the first 100 days, then gradually declined and stabilized at a relatively consistent percentage despite increasing surgical delays.

The overall percentage of medial meniscal tears per ACLR found in this study (43.7%) is higher than the reported average (33%) in studies which did not report ramp lesions,^14^^,^^30^, ^31^, ^32^^,^^42^^,^^45^, ^46^, ^47^, ^48^, ^49^, ^50^, ^51^, ^52^, ^53^, ^54^, ^55^, ^56^, ^57^, ^58^ but comparable to studies that specifically included ramp lesions.^33^^,^^59^ Giurazza et al.^33^ reported that medial meniscal tears occurred in 39% of subjects (ramp lesions = 11%), while Seil et al.^59^ reported that medial meniscal tears occurred in 41% of subjects (ramp lesions = 24%). The findings of this study suggest that some of the previous literature^30^, ^31^, ^32^, ^33^, ^34^, ^35^ has under-reported the percentage of medial meniscal tears per ACLR due to the omission of the ramp lesions. Ramp lesions are considered to be a hidden lesion of the posterior horn of the medial meniscus.^60^ Sonnery-Cottet et al.^60^ reported that only around 60% of medial meniscal tears were diagnosed when arthroscopic examination was performed through anterior visualization via an anterolateral portal. A further 40% of medial meniscal tears, mainly ramp lesions, were identified when the arthroscope was inserted into the posteromedial compartment for a more detailed examination of the posterior horn of the medial meniscus.^60^ The methodology of most publications reporting meniscal tears—including those that do not specify the percentage of ramp lesions per ACLR—does not provide information on whether posteromedial compartment arthroscopy was performed.^14^^,^^30^, ^31^, ^32^^,^^42^^,^^45^, ^46^, ^47^, ^48^, ^49^, ^50^, ^51^, ^52^, ^53^, ^54^, ^55^, ^56^, ^57^, ^58^ The absence of a detailed examination of the posterior horn of the medial meniscus within the posteromedial compartment may contribute to the under-reporting of ramp lesions and medial meniscal tears. We recommend routine examination of the posterior horn of the medial meniscus within the posteromedial compartment, including probing of the meniscus through an additional posteromedial portal, at the time of ACLR.

Similarly, the overall percentages of lateral meniscal tears per ACLR identified in this study (47.5%) is comparable to the reported values in studies that include root tears in their report^11^^,^^13^ but exceeds the average of 32.5% reported in studies that neither mentioned root tears nor specified whether some root tears were classified as radial tears.^14^^,^^30^, ^31^, ^32^^,^^42^^,^^45^, ^46^, ^47^, ^48^, ^49^, ^50^, ^51^, ^52^, ^53^, ^54^, ^55^, ^56^, ^57^, ^58^ In our cohort, root tears were present in 7.5% of knees, which was close to the pooled incidence (9.6%) from a meta-analysis of 17 studies specifically reporting the incidence of root tears.^37^ It is worth noting that there is a high variation in the reported percentage of lateral meniscal root tears per ACLR across different publications, which may indicate underdiagnosis in some studies. The percentage of root tears per ACLR reported by Ciatti et al.,^10^ Forkel et al.,^12^ and Krych et al.^13^ were 13.5%, 14%, and 17.5%, respectively. However, in certain studies, the reported percentage of root tears can be as low as 1%^34^ or 2%.^35^ While the available data preclude the adjustment of all confounding factors, the discrepancy suggests that the current literature may be underestimating the overall percentages of lateral meniscal tears per ACLR due to underreporting root tears.

Several publications in the literature aim to address the question: how does ACL deficiency lead to changes in meniscal tears over time?^31^^,^^33^^,^^35^^,^^42^, ^43^, ^44^, ^45^^,^^47^, ^48^, ^49^, ^50^, ^51^, ^52^, ^53^, ^54^^,^^54^^,^^57^^,^^58^ Regarding the percentage of meniscal tears per ACLR at the time of the initial ACL injury, it is most accurately represented by the number of meniscal tears identified during arthroscopy performed on the day of injury. However, performing ACLR on the day of injury is often not feasible due to logistical reasons, such as the availability and preferences of the patient and the surgeon. The closest estimates of the probability of meniscal tears occurring at the time of the initial injury are data obtained from ACLRs performed in the acute setting, whether defined as within 3 weeks^49^, 6 weeks^48^^,^^52^^,^^58^, or 3 months.^33^^,^^45^^,^^47^ The current study defines the acute setting as within 3 months, with observed percentages of meniscal tear per ACLR of 28.6% for medial meniscal tears, 17.1% for ramp lesions, 53.6% for lateral meniscal tears, and 10% for lateral meniscus root tears (Table 3). Notably, the percentage of meniscal tears per ACLR in this period was more variable (Figs 3, 4, and 5). Beyond this timeframe, a more stable trend in meniscal tear percentages was observed over time. Factors such as a patients’ demographic characteristics, activity levels at the time of injury, access to care, and willingness to undergo surgery may contribute the higher variability of meniscal tears in the acute setting.

While the overall percentages of medial and lateral meniscal tears per ACLR were comparable, the current study observed differences in the trends of their respective tear percentages when ACL reconstruction was performed more than 100 days after injury (Fig 3). Specifically, medial meniscal tears showed an increasing trend, whereas the percentage of lateral meniscal tear per ACLR remained unchanged. A majority of publications in the literature, including the current study, employ a similar methodology by assuming that the meniscal tears observed at the time of ACLR—performed at various intervals after injury—are representative of the additional meniscal tears that develop over time.^31^^,^^33^^,^^35^^,^^42^^,^^43^^,^^44^^,^^45^^,^^47^^,^^48^^,^^49^^,^^50^^,^^51^^,^^52^^,^^53^^,^^54^^,^^57^^,^^58^ While the rate of meniscal injury at the time of injury can be estimated from ACLRs performed in the acute setting^33^^,^^45^^,^^47^, ^48^, ^49^^,^^52^^,^^58^, assessing the rate of meniscal tears that occur between the injury and the ACLR—particularly when surgery is performed in the subacute or chronic setting—is more challenging. It is because the meniscal tears observed at the time of ACLR depend on the probability of meniscal tears present at the time of initial injury, the probability of additional meniscal tears occurring between the initial injury and the time of ACLR, and the likelihood that some of these tears have undergone spontaneous healing. If the rate of new meniscal tears that occur between the injury and ACLR exceeds the rate of spontaneous healing, a progressive increase in meniscal tears will be observed, and vice versa. The observed steady trend in the percentage of lateral meniscal tears per ACLR after 3 months from injury suggests that spontaneous healing occurs at a rate comparable to the development of new lateral meniscal tears over time. Conversely, the progressive increase in medial meniscal tear percentages over time indicates that either spontaneous healing is less readily achieved for the medial meniscus, or the medial meniscus’s role as a secondary restraint to anterior laxity in an ACL-deficient knee increases its risk of injury, or both. The question “How does ACL deficiency lead to changes in meniscal tears over time?”—specifically regarding the rate of new meniscal tears occurring between injury and ACL reconstruction—can likely only be addressed through a study that involves repeated MRIs or diagnostic arthroscopies at regular intervals, such as every 3 months, from the day of injury until the patient undergoes ACLR. The findings of this study and other publications provide only an estimate of this.

The proportion of ramp lesions among medial meniscal tears was highest during the second and third months after injury, followed by a gradual decline as the interval between injury and ACLR increased (Fig 4B), despite the overall percentage of ramp lesions per ACLR unchanged over time (Fig 4A). This apparent paradox occurs because the overall increase in medial meniscal tears was driven primarily by nonramp longitudinal tears and complex, irreparable tears, which contributed to the observed rise in medial meniscal injuries (Table 3 and Table 4). The increasing trend of medial meniscal tear percentages over time suggests that the likelihood of new meniscal tears surpasses the potential for spontaneous healing of the medial meniscus. Additionally, the rise in complex, irreparable tears over time (Table 3) indicates that some meniscal lesions, including ramp lesions, may have deteriorated and transformed into more complex morphologies. Although prior reports have documented spontaneous healing in ramp lesions and some surgeons advocate a nonoperative approach for small full-thickness ramp tears,^61^ based on the findings of this study, we recommend a more proactive strategy. Specifically, we favor repairing all full-thickness ramp lesions—regardless of size—even if they are small full-thickness tears.

**Table 4:** Distribution of Meniscal Tears According to Time: Divided Into Acute, Subacute and Chronic Settings

	Total	Acute (0–3 months)	Subacute (4–12 months)	Chronic (>12 months)	P Value
Number of knees	**731**	140	361	230	
**Knees with medial meniscal tears**	**320**	40	139	141	P < .001∗
	**43.7%**	28.6%	38.5%	61.3%	
Longitudinal tears, excluding ramp	**122**	12	59	51	P = .005∗
	**16.7%**	8.6%	16.3%	22.2%	
Horizontal tears	**2**	0	0	2	P = .11
	**0.3%**	0.0%	0.0%	0.8%	
Radial tears	**0**	0	0	0	--
	**0.0%**	0.0%	0.0%	0.0%	
Ramp lesions	**150**	24	67	59	P = .07
	**20.5%**	17.1%	18.6%	25.7%	
Root lesions	**4**	1	2	1	P = .92
	**0.5%**	0.7%	0.6%	0.4%	
Other tears	**42**	3	11	28	P < .001∗
	**5.7%**	2.1%	3%	12.2%	
**Knees with lateral meniscal tears**	**348**	75	153	120	P = .016∗
	**47.5%**	53.6%	42.4%	52.2%	
Longitudinal tears	**161**	35	78	48	P = 0.51
	**22.0%**	25.0%	21.6%	20.9%	
Horizontal tears	**14**	0	5	9	P = .018∗
	**1.9%**	0.0%	1.4%	3.9%	
Radial tears	**26**	7	7	12	P = .12
	**3.6%**	5.0%	1.9%	5.2%	
Root lesions	**55**	14	27	14	P = .28
	**7.5%**	10.0%	7.5%	6.1%	
Other tears	**92**	19	36	37	P = .09
	**12.6%**	13.6%	10%	16.1%	

Longitudinal tears include bucket-handle tears, excluding ramp lesions, while radial tears exclude root tears. Other tears include flap tears, complex tears, degenerative tears, and meniscal loss. % refers to the rates of meniscal tear in relation to the number of anterior cruciate ligament reconstructions performed at that time interval. An asterisk denotes statistical significance when the P value is <.05.

The percentage of root tears per ACLR peaked around 3 months postinjury and remained relatively unchanged thereafter (Fig 5A). The current study identified that root tears accounted for 15.8% of lateral meniscal tears. Aside from the initial 3 months, the proportion of root tears among lateral meniscal tears did not change significantly over time (Fig 5B). Increased surgical delay is not associated with an increased likelihood of root tears. The belief that root tears are associated with chronic ACL injuries is not supported by the findings of this study.

### Limitations

There were several limitations in this study. First, this study was retrospective in nature. Although most of the data were prospectively collected, this study is still prone to biases that are common in retrospective studies, particularly regarding the retrospective classification of ramp lesion and root tear subtypes based on review of operative videos and medical records.^14^^,^^20^ In addition, this study spans more than 10 years. Although the same surgeons adopted the same set of assessment criteria throughout the entire period, bias resulting from evolving surgical experience cannot be ruled out. Because the surgeons performing the ACLR were not blinded to preoperative information, including the time delay between injury and surgery, bias was likely present when they diagnosed ramp and root tears during arthroscopy. Additionally, since interobserver and intraobserver errors in the assessment of full-thickness tears was not available for analysis, observer variability may be present. Second, patients may mistake the most recent instability event for the initial injury leading to the ACL tear. This is particularly likely in cases of chronic ACL deficiency, where the initial injury may have occurred many years earlier. Recall bias from patients could have contributed to inaccuracies in estimating the true time between injury and ACLR. Third, the most common activities leading to the initial ACL injury were soccer and basketball in this study, and most patients in the current cohort were male. Because of differences in patient characteristics and activity levels, the generalizability of this study’s findings may be limited when applied to other cultural settings. Fourth, selection bias may be present. Patients were excluded if they were unclear about the date of injury, which was more likely when the injury occurred many years prior to ACLR. This could influence the observed rates and patterns of meniscal tears. Fifth, patients’ own selection bias may also have influenced the study results. For example, patients seeking earlier care might have more severe injuries, leading to a higher initial rate of meniscal injury than would otherwise be expected. Alternatively, bias can also be introduced when patients seek care for different aspects of their knee problems. For instance, if patients primarily present with symptoms of their ACL injury immediately after trauma, the observed number of meniscal tears may be relatively lower. Conversely, if they seek care for meniscal issues, higher rates of meniscal tears will be observed. All of these factors can introduce bias into the study’s outcomes, potentially affecting the study’s findings. Additionally, the situation can be further complicated by variations in the availability of medical care, patients’ socio-economic conditions, and insurance support. Consequently, the findings of this study may not be directly reproducible in centers within different healthcare systems. Sixth, the assumption that meniscal tears observed at the time of ACL reconstruction—performed at various intervals after injury—are representative of the additional tears that develop over time is an imperfect method for estimating the rate of injury progression following an ACL tear. Readers should be aware of this limitation when extrapolating the findings of this study to their clinical practice. Lastly, the definition of time intervals as 0 to 3 months, 4 to 6 months, 7 to 9 months, 10 to 12 months, 13 to 24 months, 25 to 60 months, and over 60 months has not been universally adopted, which limits the comparative potential of our results to other published literature.

## Conclusions

Aside from the initial 3 months postinjury, the percentage of medial meniscal tears per ACLR steadily increases with increasing time elapsed from injury to surgery, whereas the percentage of lateral meniscal tears per ACLR remains unchanged. Increased surgical delay is not associated with a higher chance of ramp lesions or lateral meniscus root tears observed at the time of primary ACLR.

## Disclosures

The authors (S.W., W.P.Y.) declare that they have no known competing financial interests or personal relationships that could have appeared to influence the work reported in this paper.
